# Intracranial meningioma with carcinoma tumor-to-tumor metastasis: two case reports

**DOI:** 10.2217/cns-2017-0022

**Published:** 2018-04-26

**Authors:** Jason T Pham, Ronald C Kim, Anna Nguyen, Daniela Bota, Xiao-Tang Kong, Sumeet Vadera, Frank Hsu, Jose A Carrillo

**Affiliations:** 1 Department of Neurology, University of California, Irvine Medical Center, Orange, CA 92868, USA; 2 Department of Neuropathology, University of California, Irvine Medical Center, Orange, CA 92868, USA; 3 Department of Neurological Surgery, University of California, Irvine Medical Center, Orange, CA 92868, USA

**Keywords:** adenocarcinoma, brain metastasis, breast carcinoma, cancer, meningioma, tumor-to-tumor metastasis

## Abstract

Meningiomas have been implicated as the most common primary intracranial tumor to contain tumor-to-tumor metastasis. In the following two case reports, we describe cases of adenocarcinoma and breast carcinoma that metastasized into an intracranial meningioma. The first patient was a 64-year-old man presenting to the emergency department with seizures and loss of consciousness. After a left frontal mass resection, pathology reported a heterogeneous mass consisting of a meningioma and a metastatic adenocarcinoma component. The second patient was a 63-year-old woman presenting with significant vision problems and unstable gait. After a right frontal mass resection, pathology reported a heterogeneous mass consisting of a meningioma and a metastatic breast carcinoma component. Possible explanations for the development of the tumor-to-tumor metastasis are described.

**Practice points**

- Tumor-to-tumor metastasis in meningiomas is a rare phenomenon that has been recorded <100-times by the criteria specified by Campbell.

**Case reports**

- One patient summarized suffered from tumor-to-tumor metastasis from an adenocarcinoma that may be found in the GI tract, pancreatic, biliary or lungs. Our other patient summarized suffered from tumor-to-tumor metastasis from a breast carcinoma.

**Discussion**

- Pathophysiological mechanisms of meningiomas including significant hypervascularity, increased cerebral perfusion and low metabolic activity provide a nutrient-rich environment for metastases.
- Studies suggest that primary breast neoplasms are more likely to metastasize to meningiomas due to increased estrogen and progesterone receptors found on meningiomas to mediate cell–cell interactions.
- While the most common tumor-to-tumor intracranial metastases are from primary breast and lung neoplasms, the phenomenon itself appears stochastic.
- Although tumor-to-tumor metastasis is diagnosed after surgery and biopsy, studies implicate that magnetic resonance spectroscopy and perfusion MRI superimposed with MRI scans may assist in determining a diagnosis noninvasively.
- Patients affected by tumor-to-tumor metastasis may suffer from differential diagnostic symptoms than those commonly presented with only meningiomas.

Despite the innate immune responses from resident microglia, astrocytes and infiltrating T cells, the CNS is susceptible to many different types of primary neoplasms and metastases [1]. The Central Brain Tumor Registry of the United States emphasized the rarity of CNS tumors by suggesting an incidence of 22.36 cases per 100,000. Of CNS tumors, aggressive non-neural metastases are the largest contributors with an incidence of 10 cases per 100,000 [2–4]. On the other hand, meningiomas are the most common intracranial tumors with an incidence of 7.61 cases per 100,000 [5,6].

While both types of tumors can simultaneously exist in distinct parts of the brain, meningiomas have been found to experience tumor-to-tumor metastasis [7,8]. This rare phenomenon occurs when one tumor metastasizes into another [9]. Although rare, this pathological finding has been recognized and documented within the last century [10]. Specifically, meningiomas have been described as the most common intracranial tumor to host metastases [9,11]. Malignant cells from renal, prostate, hematopoietic, gastrointestinal, breast and lung tumors have been documented to metastasize into meningiomas, with breast and lung tumors to be the most common primary sites [9,12,13]. The Surveillance, Epidemiology, and End Results program indicates the incidence of primary breast tumors as 124.9 cases per 100,000, while the incidence of primary lung tumors as 55.8 cases per 100,000 [14,15]. With the incident rates of brain tumors, these statistics highlight the possibility of coexisting primary tumors and metastases. However, not all CNS metastases result in a tumor-to-tumor diagnosis [9,16].

Tumor-to-tumor metastases could be mistaken for collision tumors, which are two separate neoplasms adjacent to one another [9,17]. Instead, tumor-to-tumor metastasis occurs when primary tumors are anatomically and pathologically contiguous. The criteria delineated for the diagnosis of tumor-to-tumor metastasis are: at least two primary tumors must exist; the host tumor must be a true neoplasm; the metastatic focus must show established growth inside the host tumor, and not be of contiguous growth; the host tumor cannot be a lymph node involved in leukemia or lymphoma [9,16]. According to this schema, there have been fewer than 100 recorded cases of tumor-to-tumor intracranial metastases [10,18].

Two separate cases of adenocarcinoma and breast carcinoma yielding metastasis into intracranial meningioma are presented.

## Case 1

**Figure 1.**
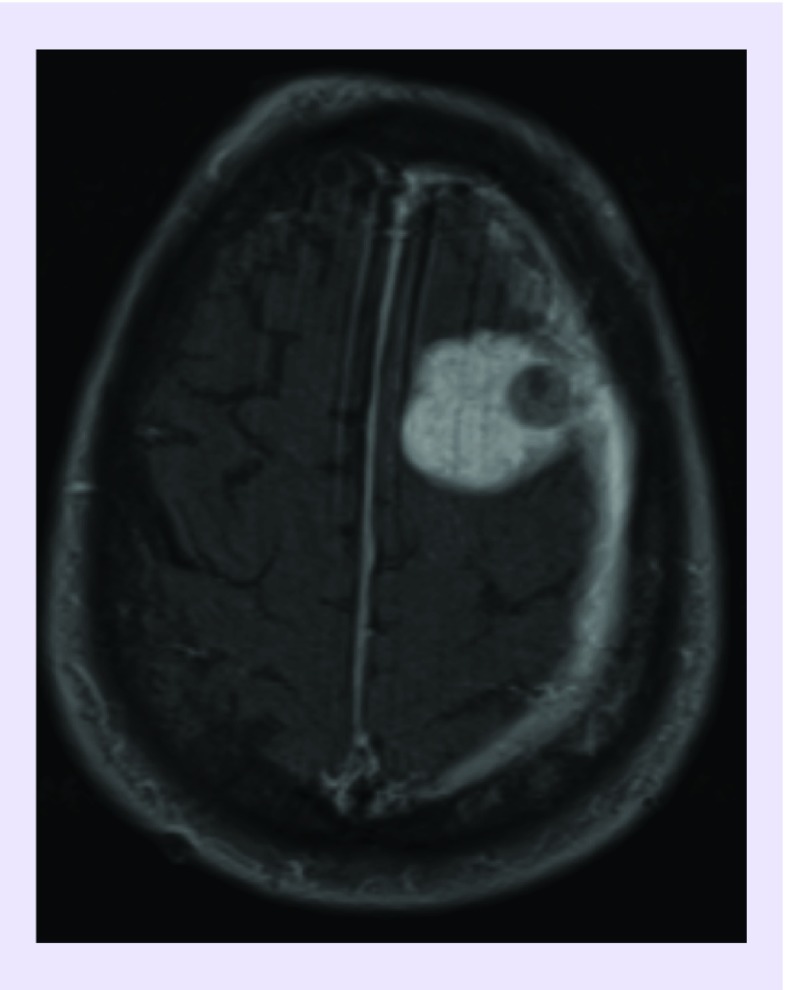
**Axial T1 contrast-enhanced MRI.** An axial T1 contrast-enhanced MRI image demonstrates a large left frontal enhancing mass. Within the lesion is a small, round nonenhancing mass.

A 64-year-old man presented with chronic episodes of seizures and recent loss of consciousness. The patient's past medical history is notable for a diagnosed lung tumor 2 years prior without any medical treatment. On admission, an MRI brain scan revealed a left frontal mass (Figure 1).

**Figure 2.**
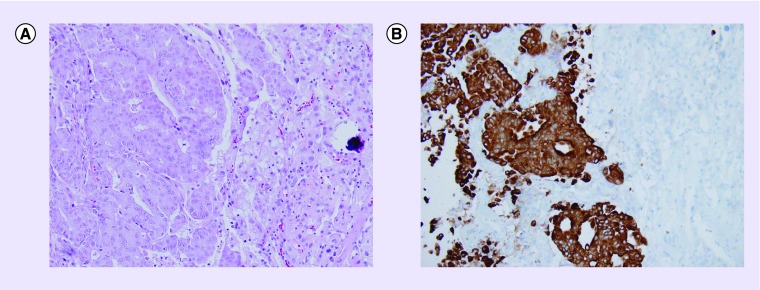
**Metastatic adenocarcinoma and meningioma.** The debulking of the left frontal mass was received in an aggregate. It consisted of irregular pink focally hemorrhagic soft tissue fragments measuring up to 3.3 cm in aggregate. **(A)** On microscopy, it showed a moderately differentiated metastatic adenocarcinoma in collision with an associated WHO grade 1 meningioma with a psammoma body. **(B)** The adenocarcinoma was immunoreactive for CK7 and the meningioma lacked immunoreactivity for CK7. The meningioma component was positive for SSTR2 and PR with a proliferation index of 1–2%, confirming the low grade of the meningioma.

The patient underwent a left craniotomy for a left frontal mass resection. Pathology consisted of a moderately differentiated metastatic adenocarcinoma in association with a WHO grade I meningioma (Figure 2). The metastatic adenocarcinoma component was strongly positive for CK7 and MUC5, weakly positive for STAB2, CDX2 and CK17, and negative for CK20, TTF1, napsin, NKX3.1 and P501S. The meningioma component was strongly positive for SSTR2 and PR. Ki-67 proliferation index was 1–2% in the meningioma and 30% in the metastatic adenocarcinoma component. Unfortunately, an EMA staining was not conducted due to limited samples collected. The origin of metastases was unknown, but potential primary sites included GI tract, pancreatic biliary and lungs.

Postoperatively, the patient did well. Neurological examination of the patient did not reveal cognitive deficits. Cranial nerves and mental status examination were normal. The patient developed diminished sensory to light touch and temperature in the left upper extremities and diminished sensory to vibration in the lower extremities. The patient showed Romberg sign and ataxic tandem gait.

The patient was referred to radiation oncology for stereotactic radiosurgery and hematology oncology for work up and management of his primary cancer. Unfortunately, this patient was lost to follow-up.

## Case 2

**Figure 3.**
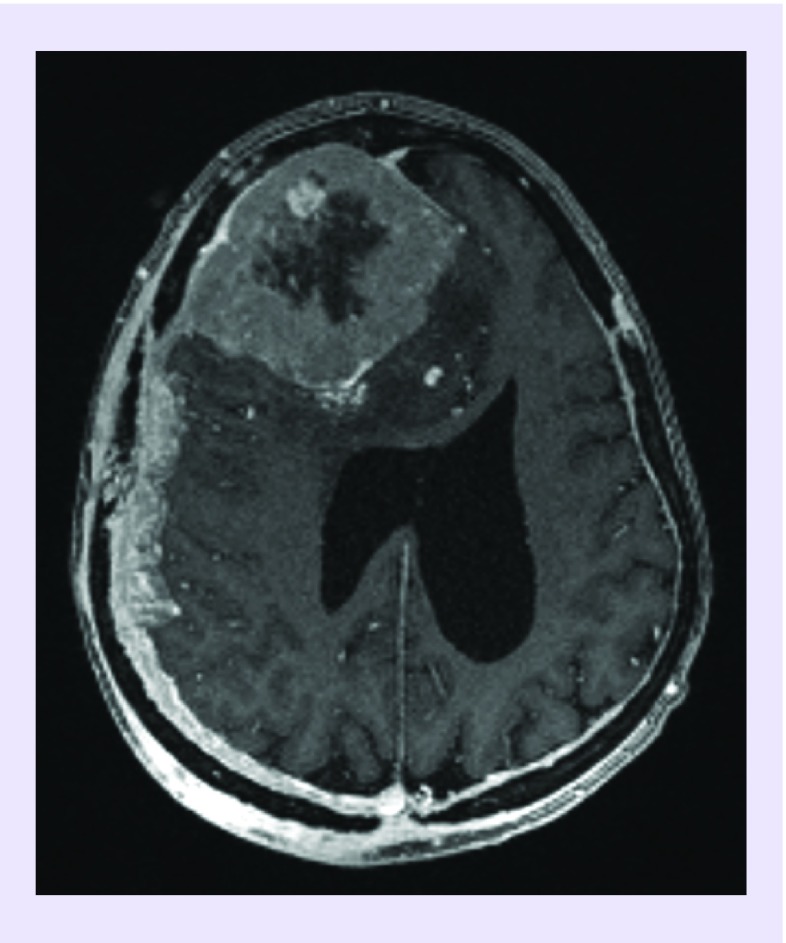
**Axial T1 contrast-enhanced MRI.** An axial T1 contrast-enhanced MRI image demonstrates large right frontal enhancing dural-based mass. Within the lesion is a centrally located small, round, homogeneously enhancing mass.

A 63-year-old woman was admitted with significant vision problems and unstable gait. The patient previously saw an oncologist for a modified Bloom–Richardson grade III ER+/PR+/HER2-negative ductal carcinoma that had metastasized to the patient's axillary lymph node. Breast lumpectomy and axillary lymph node removal was performed, followed by chemotherapy and hormone therapy. There was no initial brain MRI screening at the time. After 3 years, she presented with increasing amount of falls and vision loss. A cerebral MRI found a right frontal brain mass (Figure 3).

**Figure 4.**
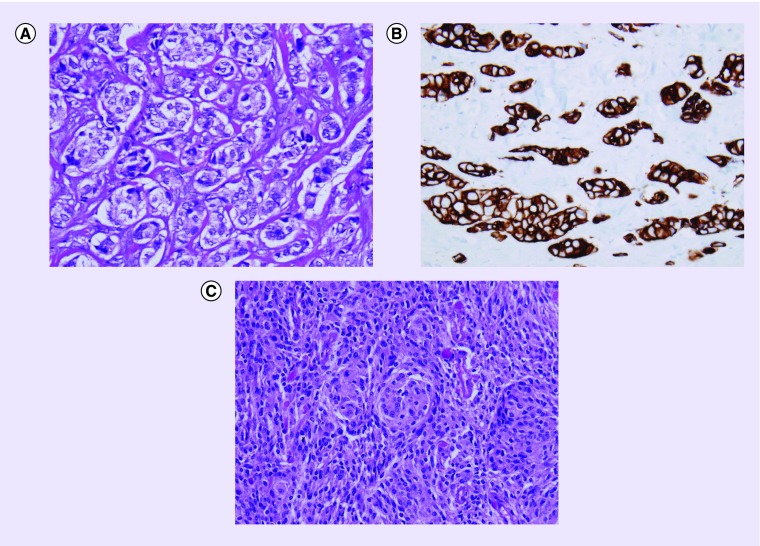
**Metastatic carcinoma and meningioma.** The mass lesion consisted of two separate and distinct neoplasms in close juxtaposition, namely, metastatic carcinoma and anaplastic (WHO grade 3) meningioma. **(A)** Extracranial lesion consisting of gelatinous masses of tissue characterized histologically by neoplastic tissue show epithelial cells with gland formation. **(B)** Extracranial lesion consisting of immunoreactivity for CK7, ER, and PR, as well as a small focus of non-gland-forming tumor showing moderate cellularity with whorl formation and lack of immunoreactivity for CK7. **(C)** Intradural tumor consisted of non-gland-forming tumor showing whorl formation and moderate cellularity, immunoreactivity for SSTR2, lack of immunoreactivity for CK7, and a proliferation index of 10–12%.

The patient underwent a right subtotal craniotomy for tumor resection and biopsy. The right frontal mass excision consisted of a metastatic primary breast neoplasm and an anaplastic meningioma WHO grade III (Figure 4). The extracranial lesion was CK7, ER- and PR-positive, while CK20, HER2 and HER2 dual *in situ* hybridization (ISH)-negative. The meningioma was CK7-, ER- and AE1-/AE3-negative, while SSTR2-, PR- and CD68-positive. Ki-67 proliferation index was approximately 10–12% for the meningioma. Unfortunately, an EMA staining was not conducted due to limited samples collected.

Postoperatively, the patient was treated with radiation to prevent recurrence and received a ventriculoperitoneal shunt due to hydrocephalus and findings of leptomeningeal disease. The patient continued to suffer from vision impairment and decreased extraocular muscle movement. Otherwise, neurological examination of the patient did not reveal cognitive deficits. Mental and physical examination was normal.

We followed up with the patient for biweekly intrathecal liposomal cytarabine treatment. The patient was prescribed dexamethasone in association with the intrathecal treatment. Although further molecular characterization of the primary and metastatic tumor sites and additional follow-ups would have provided more detailed information for this case, the patient has since passed and neither is no longer possible.

## Discussion

Tumor-to-tumor metastasis to meningioma is a rare CNS phenomenon first recorded in the 1930s by Fried [19]. Since, there have been <100 intracranial cases recorded under specific criteria delineated by Campbell and Pamphlett [9,10,16]. Researchers have been attempting to discover definite causes on whether a primary neoplasm will undergo tumor-to-tumor metastasis by examining the intrinsic nature of meningiomas and the genetic components of neoplasms.

There are various pathophysiological mechanisms that suggest the commonality of tumor-to-tumor metastasis to meningiomas than other intracranial neoplasms. Meningiomas are known to have significant hypervascularity, increased cerebral perfusion and low metabolic activity. In combination with its slow growth and indolent nature, these suggest that meningiomas provide a nutrient-rich environment for metastasis [20–22]. Meningiomas also contain cell–cell adhesion molecules (CAMs), which commonly assist with signal transduction, cell growth and cell–extracellular matrix adhesion [23]. E-cadherin, a type of cell–cell adhesion molecule, is commonly found in both primary carcinomas and their metastases, which may implicate increased metastases in comparison to other neoplasms [24,25]. In addition, estrogen and progesterone receptors found on meningiomas mediate cell–cell interactions, specifically with breast tumor metastases [11,26].

Breast carcinomas are known to be the most frequent primary neoplasm to have tumor-to-tumor metastasis with meningiomas. While our patient did not have correlative mutations with the meningioma, there are other factors that may be involved. Breast carcinoma and meningiomas are conspicuous to have considerable amounts of estrogen and progesterone receptors [11,25]. Specifically, meningiomas may have progesterone and estrogen receptors as high as 90 and 30%, respectively. Breast neoplasms may have progesterone and estrogen receptors as high as 79.6 and 66.8%, respectively [25,27]. This is consistent with the increased prevalence of tumor-to-tumor metastasis of breast carcinoma to meningioma. Despite these posits from previous literature, the rarity of metastasis suggests it may be a stochastic phenomenon [11].

The rarity of intrameningeal metastasis may be attributed to its difficulty in detecting without surgery or biopsy. The current use of neuroimaging such as a CT scan or an MRI scan cannot rule out a metastasis due to lack of specificity. Therefore, many studies provide a proper diagnosis after histological examination [24]. Other literature suggests that perfusion MRIs can assist in diagnosis prior to surgery. Perfusion MRI is capable of differentiating relative blood flow and volumes of the meningioma and the metastasis, while magnetic resonance (MR) spectroscopy can determine metabolite ratios that distinguishe the meningioma and the metastasis [28–30]. Clinically, a variety of symptoms and signs will indicate if a patient develops a meningioma [31]. These include epileptic seizures, headaches, nausea and cognitive deficits depending on the size and location of the tumor. Metastasis into meningiomas may present minor differential diagnostic symptoms or increased symptoms from the meningioma [8].

## Conclusion

Tumor-to-tumor metastasis to meningioma is an infrequent phenomenon; however, there has been an increasing amount of documentation to further understand the event. Clinicians caring for patients with meningioma and other common primary neoplasms that metastasize to the CNS should be familiar with the possibility of tumor-to-tumor metastasis. Currently, the main diagnosis for intrameningeal metastasis is surgery and biopsy of the tumor. However, in recognizing possible tumor-to-tumor metastasis with clinically worsening patients who are affected by multiple primary neoplasms, physicians may determine the cause by superimposing MR spectroscopy and perfusion MRI to provide noninvasive care. We conclude that other factors are required to delineate the prognosis and diagnosis of tumor-to-tumor metastasis with methods other than biopsy in order to provide better management for our patients.
